# Extracorporeal shock wave therapy in musculoskeletal disorders

**DOI:** 10.1186/1749-799X-8-22

**Published:** 2013-07-17

**Authors:** Nikolaus BM Császár, Christoph Schmitz

**Affiliations:** 1 Department of Neuroanatomy, Ludwig-Maximilians-University, Pettenkoferstr. 11, Munich 80336, Germany

## To the Editor

We read with interest the review article by Dr. C.J. Wang concerning ‘Extracorporeal shockwave therapy in musculoskeletal disorders’ published recently in the *Journal of Orthopaedic Surgery and Research* (2012, 7:11). Despite many interesting approaches, this article unfortunately ignores some major achievements that have been made during the last years in extracorporeal shock wave therapy (ESWT) in musculoskeletal disorders:

• Wang [1] mentioned three main techniques through which shockwaves are generated: the electrohydraulic, electromagnetic, and piezoelectric principles. He contrasted them with ultrasound waves. Unfortunately, Wang [1] forgot to mention the fourth technique, the ballistic/radial principle. Notably, Wang [1] cited two randomized controlled trials (RCTs) performed with the ballistic/radial principle: (1) Gerdesmeyer et al.’s study on plantar fasciopathy [2] and (2) Rompe et al.’s study on insertional Achilles tendinopathy [3]. The study by Rompe et al. [3] was explicitly discussed as an ESWT study by Wang [1]: ‘Rompe et al. compared 25 patients treated by eccentric stretching exercises with 25 patients treated with repetitive ESWT, and the results showed that eccentric loading is inferior to ESWT in the treatment of patients with chronic recalcitrant Achilles tendinopathy’ (page 5, left column, lines 10ff in [1]).

• Given the fact that Wang [1] cited two RCTs performed with the ballistic/radial principle [2,3], one wonders why he did not cite the other RCTs that were performed with the same principle and showed the efficacy and safety of ESWT in muskuloskeletal disorders according to the principles of Evidence Based Medicine (EBM) Levels I (evidence obtained from at least one properly designed randomized controlled trial) and II (evidence obtained from a well-designed cohort or case-control analytic studies, preferably from more than one center or research group) (EBM levels established by the United States Preventive Services Task Force; http://www.uspreventiveservicestaskforce.org) [4-11].

• Wang’s [1] list of ESWT devices approved by the United States Food and Drug Administration (FDA) is incomplete. Actually, the following ESWT devices obtained Premarket Approval (PMA) from the FDA as Class III orthopedic lithotripsy devices and were re-classified as Class III Generators, Shock Wave, For Pain Relief (Product Code NBN) in the spring 2009: (1) OssaTron (HealthTronics, Inc., Marietta, GA, USA), PMA no. P990086 issued on October 12, 2000 to treat chronic heel pain; (2) Epos Ultra (Dornier Medical Systems, Inc., Kennesaw, GA, USA), PMA no. P000048 issued on January 15, 2002 for the treatment of chronic plantar fasciitis for patients with symptoms of plantar fasciitis for 6 months or more and a history of unsuccessful conservative therapy; (3) SONOCUR Basic (Siemens Medical Solutions, Inc., Iselin, NJ, USA), PMA no. P010039 issued on July 19, 2002 for the treatment of pain due to tennis elbow; (4) Orthospec Extracorporeal Shock Wave Therapy (Medispec, Ltd, Germantown, MD, USA), PMA no. P040026 issued on April 1, 2005 for the treatment of proximal plantar fasciitis with or without heel spur in patients 18 years of age or older; (5) Orbasone Pain Relief System (Orthometrix, Inc., White Plains, NY, UAS), PMA no. P040039 issued on August 10, 2005 to relieve heel pain (proximal plantar fasciitis); and (6) Swiss DolorClast (EMS Electro Medical Systems; Dallas, TX, USA), PMA no. P050004 issued on May 8, 2007 to treat heel pain associated with chronic proximal plantar fasciitis.

The Swiss DolorClast is a device that makes use of the ballistic/radial principle and was used in the studies mentioned above [2-11], including the studies by Gerdesmeyer et al. [2] and Rompe et al. [3] cited by Wang [1] as ESWT studies. In summary, Wang’s [1] review appears incomplete and should be revised.

Author’s response

 Ching-Jen Wang

In response to Dr. Christoph Schmitz and Nikolaus Császár’s letter concerning my published article, the inclusion of ballistic/radial principle shockwave as the fourth shockwave technique remains controversial. Some considered the ballistic/radial shockwave as one form of shockwave technique even though the working mechanism may be different from other shockwave devices. The articles cited in an article is dependent on the time the article was written, and they might not be complete as mentioned.

The lists of FDA-approved shockwave devices change from time to time. The information in the article was provided based on the author’s best knowledge at that time, and it is for reference only. I hope this response has answered your questions satisfactorily. Thank you for the interest in this article.

## Competing interests

CS serves as a paid consultant for and receives benefits from Electro Medical Systems S.A. (Nyon, Switzerland), the manufacturer and distributor of the Swiss Dolorclast radial shock wave device. Accordingly, CS has received benefits for personal use from a commercial party related directly or indirectly to the subject of this article. However, NC and CS declare that they have not received any honoraria or consultancy fee in writing this manuscript. No benefit was received or will be received directly or indirectly from a commercial party related to the performance of this study.

## Authors’ contributions

NC and CS participated in the study with the responsibility in protocol drafting, reference search, data collection and analysis, manuscript writing, and final proofing of the manuscript. Both authors read and approved the final manuscript.
